# Nanoparticle to Nanoparticle Bioorthogonal Detection of Atherosclerosis

**DOI:** 10.1021/acsami.5c08945

**Published:** 2025-10-06

**Authors:** María Muñoz-Hernando, Paula Nogales, Andrea Rodríguez-San Pedro, Marta Ibañez, Miguel Ángel Morcillo, Leticia González-Cintado, Jacob F. Bentzon, Fernando Herranz

**Affiliations:** † Grupo de Nanomedicina e Imagen Molecular, Instituto de Química Médica (IQM/CSIC), Juan de la Cierva 3, 28006 Madrid, Spain; ‡ 38799 Centro Nacional de Investigaciones Cardiovasculares, CNIC, Melchor Fernández-Almagro 3, 28029 Madrid, Spain; § Department of Clinical Medicine, Aarhus University, 8200 Aarhus, Denmark; ∥ Unidad de Aplicaciones Médicas de las Radiaciones Ionizantes, 54457 Centro de Investigaciones Energéticas, Medioambientales y Técnicas (CIEMAT), 28040 Madrid, Spain; ⊥ CIBER Enfermedades Respiratorias (CIBERES), Melchor Fernández-Almagro 3, 28029 Madrid, Spain

**Keywords:** atherosclerosis, bioorthogonal chemistry, nanomaterials, molecular imaging, ^68^Ga, iron oxide

## Abstract

*In vivo* identification and characterization of atherosclerosis is a promising approach for the development of novel therapies and personalized treatments. Among the methods for this *in vivo* identification, the use of pretargeted imaging shows very large probe uptakes and excellent selectivity. However, this approach relies on the use of antibodies which may limit their usability. In this study, we introduce a pretargeting imaging approach for atherosclerosis detection using PET that only employs nanomaterials. Here we develop the concept of nanoparticle-to-nanoparticle pretargeted imaging for atherosclerosis. Sphingomyelin solid lipid nanoparticles (sphNP) functionalized with trans-cyclooctene (TCO) were used as targeting agents and accumulated in atherosclerotic plaques. This was followed by the injection of ^68^Ga-doped nanotracers functionalized with tetrazine ([^68^Ga]­Ga-IONP-Tz), which binds to the accumulated sphNP-TCO via bioorthogonal click chemistry. *In vivo* PET imaging showed clear uptake in the aortic arch of mice receiving the full pretargeting approach, while the control groups showed no significant signal. This nanoparticle-based pretargeting strategy enables noninvasive PET imaging of atherosclerosis without using antibodies. This approach expands the use of bioorthogonal imaging and may have potential for targeted drug delivery to atherosclerotic plaques.

## Graphical abstract

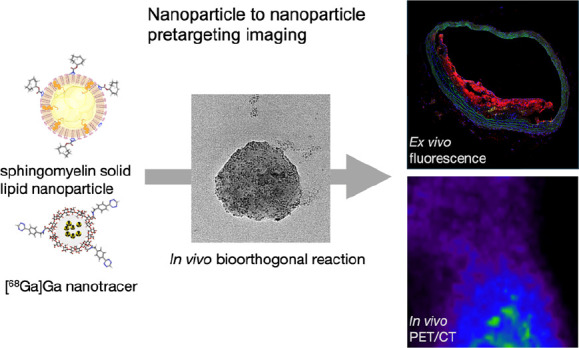

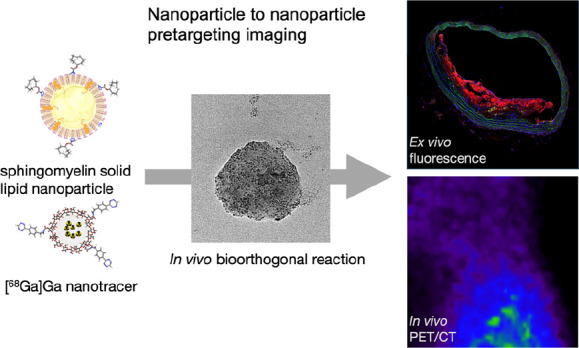

## Introduction

Bioorthogonal chemistry refers chemical reactions that can occur in living systems without interfering with native biochemical processes.^1^ It has emerged as a powerful tool in chemical biology and for the development of nanoparticle-based imaging agents.^2^ Pretargeting methods involve the use of bioorthogonal chemistry for complementary *in vivo* labeling of a targeting agent (typically an antibody or a peptide) with a tracer (typically an NP or a radioisotope) and have been recently used in different imaging modalities.^3,4^ During a pretargeted molecular imaging approach, the targeting agent is first injected and allowed to accumulate, following which the tracer is administered and selectively accumulates at the site of the bioorthogonal reaction. This method is particularly appealing for PET because it allows the use of short-half-life radioisotopes to detect targeting agents with long biodistribution times.

Atherosclerosis is a chronic inflammatory disease affecting the arterial wall and is characterized by inflammation in the intimal layer. The inflammation is mainly due to the intimal retention and accumulation of cholesterol-rich apolipoprotein B (ApoB) containing lipoproteins, predominantly low-density lipoproteins (LDLs), which acquire molecular patterns that activate innate and adaptive immune cells.^5^ The continuous influx of atherogenic lipoproteins fuels a chronic low-level inflammatory state that ultimately drives plaque development. Multiple other risk factors accelerate the disease process, including arterial hypertension, diabetes mellitus, and genetic predisposition, making atherosclerosis a complex multifactorial disease.

Several enzymes have been shown to be important in retaining LDLs in the intimal and developing plaque, including lipoprotein lipase (LpL), secretory sphingomyelinase (SMase), and secretory phospholipase A2 (sPLA2).^6^ LpL acts as a bridge between proteoglycans and lipoproteins, increasing their binding affinity and thus favoring retention.^7^ s-SMase hydrolyses sphingomyelin (sph) present on the surface of atherogenic LDLs into ceramide, promoting lipoprotein aggregation. LDL aggregation further promotes retention by increasing proteoglycan binding affinity and impairing the diffusion of large aggregates back into the lumen.^8^ Finally, sPLA2, similar to SMase, hydrolyses phosphatidylcholine present in lipoproteins, making them more susceptible to aggregation.^9^

We have recently shown that these mechanisms of LDL retention can be hijacked to drive nanoparticle accumulation in atherosclerotic plaques.^10,11^ Of particular interest for the present work is that we found that iron-oxide-based sph nanomicelles could be aggregated by sphingomyelinase *in vitro* and that they accumulate in sphingomyelinase-expressing regions of atherosclerotic plaque in mice.^10^

Here, we synthesized sphingomyelin solid lipid nanoparticles (sphNP), formed by a solid lipid core matrix composed of sphingomyelin molecules and stabilized using cholesterol. This is a more translational approach that retains the ability of the particles to be accumulated in atherosclerotic lesions by SMase, which removes the zwitterionic head of sphingomyelin responsible for colloidal stability. Our aim was to combine the selective targeting of our lipidic nanoparticles to atherosclerotic lesions with the diagnostic power of imaging.

While direct or indirect radiolabeling of lipidic nanoparticles is feasible, with numerous successful examples demonstrating various methods for incorporating different radioisotopes into nanoparticles, we sought to investigate an alternative approach. We opted to examine the utilization of a pretargeted strategy, which is not commonly employed with lipid nanoparticles. Moreover, we devised a novel pretargeted method that diverges from conventional designs. Rather than employing an antibody for biological targeting, as is typically done, we utilized sphNP as the biological targeting agent and ^68^Ga-doped citrate-coated iron oxide ([^68^Ga]­Ga-IONP), previously developed by our team,^12^ as the tracer. This innovative approach introduces the concept of nanoparticle-to-nanoparticle pretargeting imaging.

Among the reactions showing bioorthogonal features, Inverse Electron-Demand Diels–Alder (IEDDA) cycloaddition or tetrazine ligation is best suited for *in vivo* imaging.^13^ IEDDA cycloaddition is a highly efficient and selective conjugation that involves the reaction between a tetrazine and a strained alkene, such as trans-cyclooctene (TCO), to yield a stable, covalent bond in a very fast manner. This reaction can be carried out under physiological conditions and has been shown to be highly selective and fast, enabling the labeling of biomolecules and nanoparticles with minimal interference.^14^ Therefore, different research groups, including ours, have used this reaction to develop nanoparticle-based imaging probes for targeted molecular imaging, and have demonstrated their functionality in models of cancer and cardiovascular disease, among others.^15–17^ Given these advantages, this reaction was chosen for the NP-to-NP approach. Therefore, we covalently attached TCO molecules to sphNP for use as a targeting agent, allowing their accumulation in atherosclerotic lesions *in vivo*. Subsequently, [^68^Ga]­Ga-IONP functionalized with tetrazine ([^68^Ga]­Ga-IONP-Tz) were used as nanotracers. This nanotracer is routinely used for *the in vivo* molecular imaging of various pathologies and models.(Scheme 1)^18–21^

**Scheme 1:**
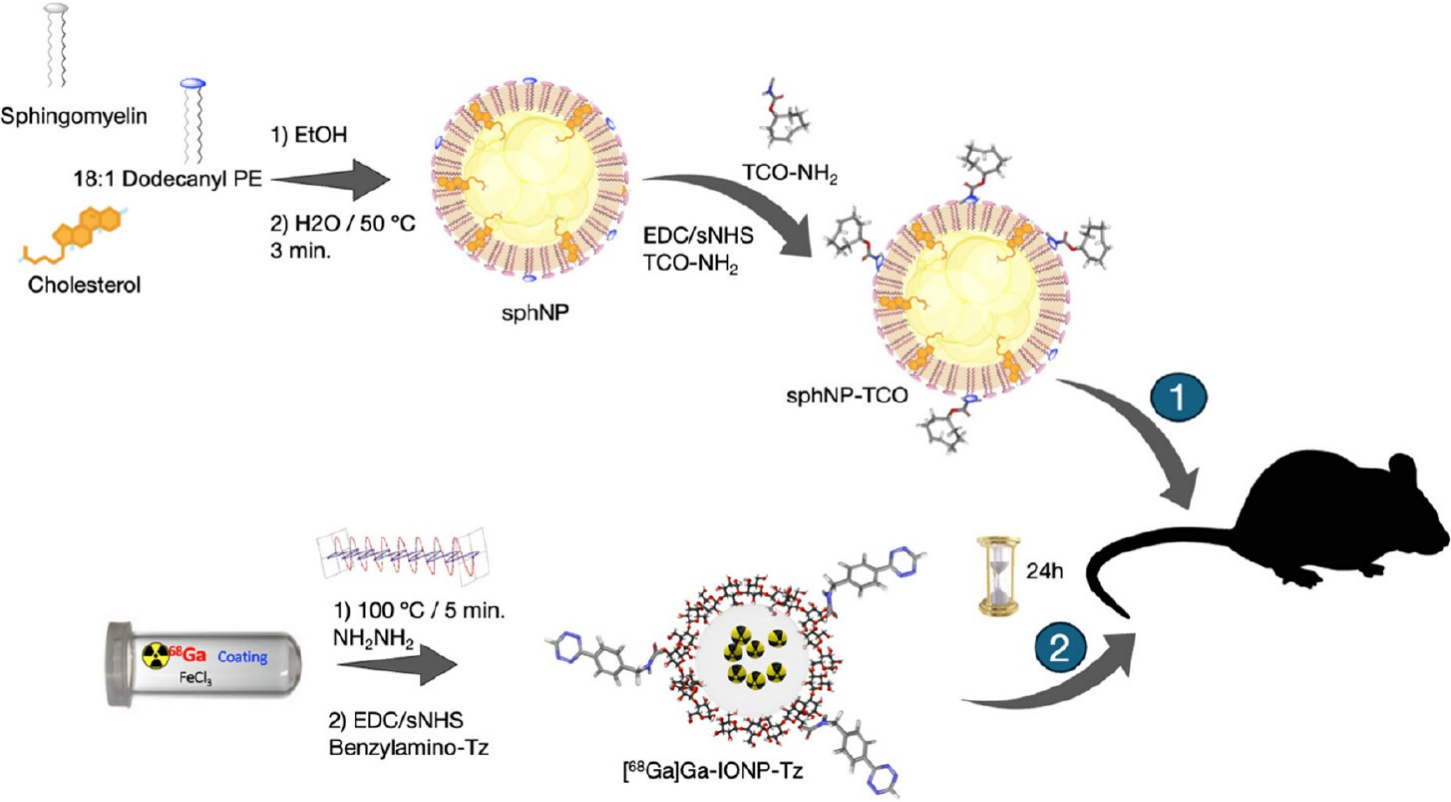
Schematic Representation of the Bioorthogonal Approach Used in This Study^a^

a First, sphNP were synthesised by mixing sphingomyelin (sph), cholesterol, and 18:1 dodecanyl PE in ethanol, followed by injecting the solution into water at 50 °C and stirring for 3 min sphNP were purified, and TCO moieties were introduced by covalent coupling with TCO-NH_2_. Purified sphNP-TCO were intravenously injected and allowed for 24 h *in vivo* distribution. Then, [^68^Ga]­Ga-IONP were synthesised in a one-step microwave-driven process, tetrazine groups were covalently added to the surface, and the radiotracer was intravenously injected and allowed for distribution prior to PET and histological analysis.

## Results and Discussion

### Synthesis and Characterization of Sphingomyelin-Solid Lipid Nanoparticles

sphNP were produced using a solvent injection method. First, sph and cholesterol were dissolved in ethanol. Subsequently, they were rapidly injected into water at 50 °C under constant magnetic stirring. After 3 min, the resulting NP were purified by size exclusion chromatography to remove any excess lipids. The same protocol was used to produce fluorescent sphNP except that 0.1 mg of the fluorophore DilC18(5) was added to the ethanol mixture prior to injection into aqueous media. The nanoparticles showed a narrow size distribution with a polydispersity index (PDI) of 0.18, hydrodynamic size of 145 ± 2 nm, and ζ-potential value of −10 ± 2 mV. Different sphNP batches were reproducibly synthesized (Figure 1a). Nanoparticles showed very good stability over time; only minimal changes in their hydrodynamic size were observed between time points for at least 72 h post-NP synthesis (Figure 1b). Negative staining transmission electron microscopy (TEM) showed a homogeneous size distribution for all sphNP samples. We measured several sphNP, *N* = 100 for each synthesis, and their size was uniform for all three batches, approximately 43 ± 6 nm in core size (Figures 1d and S1).

**Figure 1:**
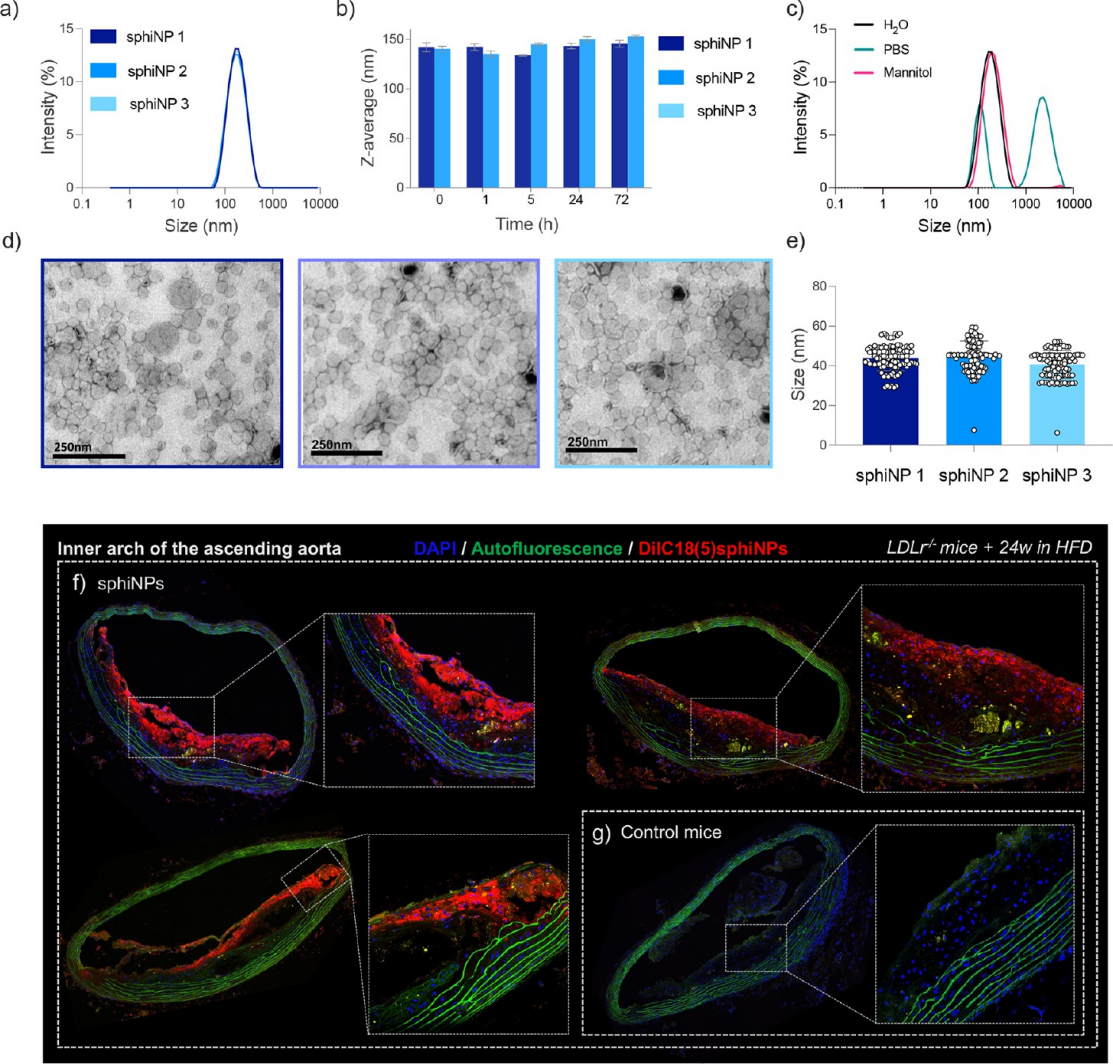
Physicochemical characterization of sphNP. (a) Hydrodynamic size distribution of sphNP determined by DLS (mean distribution of three different batches). (b) Hydrodynamic size (z-average, mean ± SD) of two different sphNP solutions in H_2_O from *t* = 0 to *t* = 72 h (slope of the linear trend with time not significantly different from 0 *p* = 0.24). (c) Hydrodynamic size distribution of sphNP in H_2_O, PBS, and mannitol, measured 1 h after mannitol addition (mean distribution of three different batches). (d) TEM images of three different sphNP solutions, negatively stained with 2% uranyl acetate for lipid visualization; scale bar, 250 nm. (e) sphNP size (*N* = 100) measured from the TEM images in (d), corresponding to three different sphNP batches (each point corresponds to a measured NP). Representative confocal microscopy images of sections of the inner arch of the ascending aorta of mice injected with (f) fluorescent sphNP (150 μL) or (g) noninjected controls. DAPI (blue), autofluorescence (green), and DilC18(5)­sphNP (red). Scale bars are 200 μm.

Once the stability of the sphNP and the reproducibility of their synthesis method were demonstrated, we proceeded to make them suitable for *in vivo* injection. For this purpose, the medium was changed from H_2_O to 1× PBS. However, sphNP did not remain stable in PBS, rapidly showing large aggregates (Figure 1c). Therefore, as an alternative, a small amount of mannitol (55 mg/mL) was diluted into the sphNP suspension, and its stability was measured 1 h after addition. Mannitol, which is commonly used for nanoparticle injection,^22^ acts as an osmoprotectant, mitigating the risk of osmotic stress and ensuring safe and effective NP delivery. The stability results indicated that the sphNP remained stable in mannitol, showing homogeneous hydrodynamic size distributions, similar to the size distributions in water (Figure 1c).

### *Ex Vivo* Confocal Microscopy of sphNP Accumulation

After fully characterizing the physicochemical properties of sphNP, we performed *in vivo* experiments. Given the sphNP composition, which resembles that of LDL particles, and our previous experience with iron-oxide based sph nanomicelles, we expected them to accumulate in atherosclerotic lesions. To confirm this, we performed a pilot fluorescence experiment in which sphNP accumulation was assessed using *ex vivo* confocal microscopy. Fluorescently labeled sphNP were administered by tail vein injection in atherosclerotic Ldlr^*–/–*^ mice that had been fed a HFD for 24 weeks and were allowed to circulate and accumulate. After 24 h, the mice were euthanised by exsanguination and perfusion-fixed, and their aortas were extracted and sectioned for *ex vivo* confocal microscopy.

Figure 1f,g show representative confocal microscopy images of sections of the inner arch of the ascending aorta from Ldlr^*–/–*^ mice injected with fluorescent-labeled sphNP and from noninjected controls. The results showed a clear accumulation of sphNP in the atherosclerotic plaque, with a larger accumulation in the plaque shoulders and plaque cells closer to the lumen. No signal was observed in the aortic sections of noninjected mice (Figure 1g).

These results validated our hypothesis by showing that sphNP accumulate *in vivo* in atherosclerotic lesions, making them a potential nanoparticle-based imaging agent for the detection of atherosclerosis. However, they can only be detected using optical imaging techniques, which have inherent limitations for the noninvasive detection of atherosclerosis owing to the limited penetration depth of light in biological tissues. Therefore, we designed a strategy to use sphNP with molecular imaging techniques, such as PET, which have proven to be more successful for the noninvasive characterization of atherosclerosis.^24^

### Atherosclerosis Detection *In Vivo* Using a Nanoparticle-to-Nanoparticle Pretargeting Approach for PET Imaging

To produce trans-cyclooctene (TCO)-functionalized sphNP, a multiple-step protocol was used. First, sphNP with carboxyl groups on the surface (sphNP-COOH) were prepared using the solvent injection method. A similar protocol to that used to produce sphNP was followed, except that 18:1 Dodecanyl PE (a headgroup-modified phospholipid containing carboxylate groups) was added to the organic phase prior to its injection into the aqueous media. Upon completion of this step, (i) the carboxyl groups of sphNP-COOH were activated in the presence of EDC and sulfo-NHS, and (ii) the TCO moiety was incorporated into the sphNP-COOH surface through amide formation between the added TCO-amine molecules and carboxyl groups, forming sphNP-TCO.

To evaluate the colloidal properties of the samples, their hydrodynamic sizes were measured by dynamic light scattering (DLS). Both samples showed narrow size distributions with hydrodynamic sizes of 103 ± 3 nm (PDI = 0.22 ± 0.03) for sphNP-COOH and 149 ± 5 nm (PDI = 0.25 ± 0.025) for sphNP-TCO (Figure 2a). Moreover, the synthetic method showed high reproducibility, with mean hydrodynamic sizes showing a very narrow distribution over six independent syntheses (Figure 2b). It is worth noting that the hydrodynamic size of sphNP-COOH was smaller than that of sphNP. This effect could be due to the incorporation of carboxyl groups on the surface of the nanoparticles, which are ionised in water, creating electrostatic repulsion between NP, thereby increasing their colloidal stability and resulting in a smaller effective hydrodynamic size. In addition, there was a change in the hydrodynamic size before and after the conjugation of SLNP with TCO, which could indicate the successful incorporation of the TCO molecules into the surface of the sphNP. The surface charge was also assessed using DLS. ζ-Potential measurements showed that functionalization with TCO produced a small shift in the surface charge values, from a mean of −23 mV for sphNP-COOH to −11 mV for sphNP-TCO (*N* = 4) (Figure 2c). This could be due to a reduction in the number of free carboxyl groups on the surface, further indicating the successful incorporation of the TCO moiety into the sphNP. Notably, the sphNP-TCO surface charge was close to that of sphNP, which did not contain free COOH groups on its surface (Figure 2c). Additional tests were performed to confirm the presence of TCO molecules as described in the following sections.

**Figure 2:**
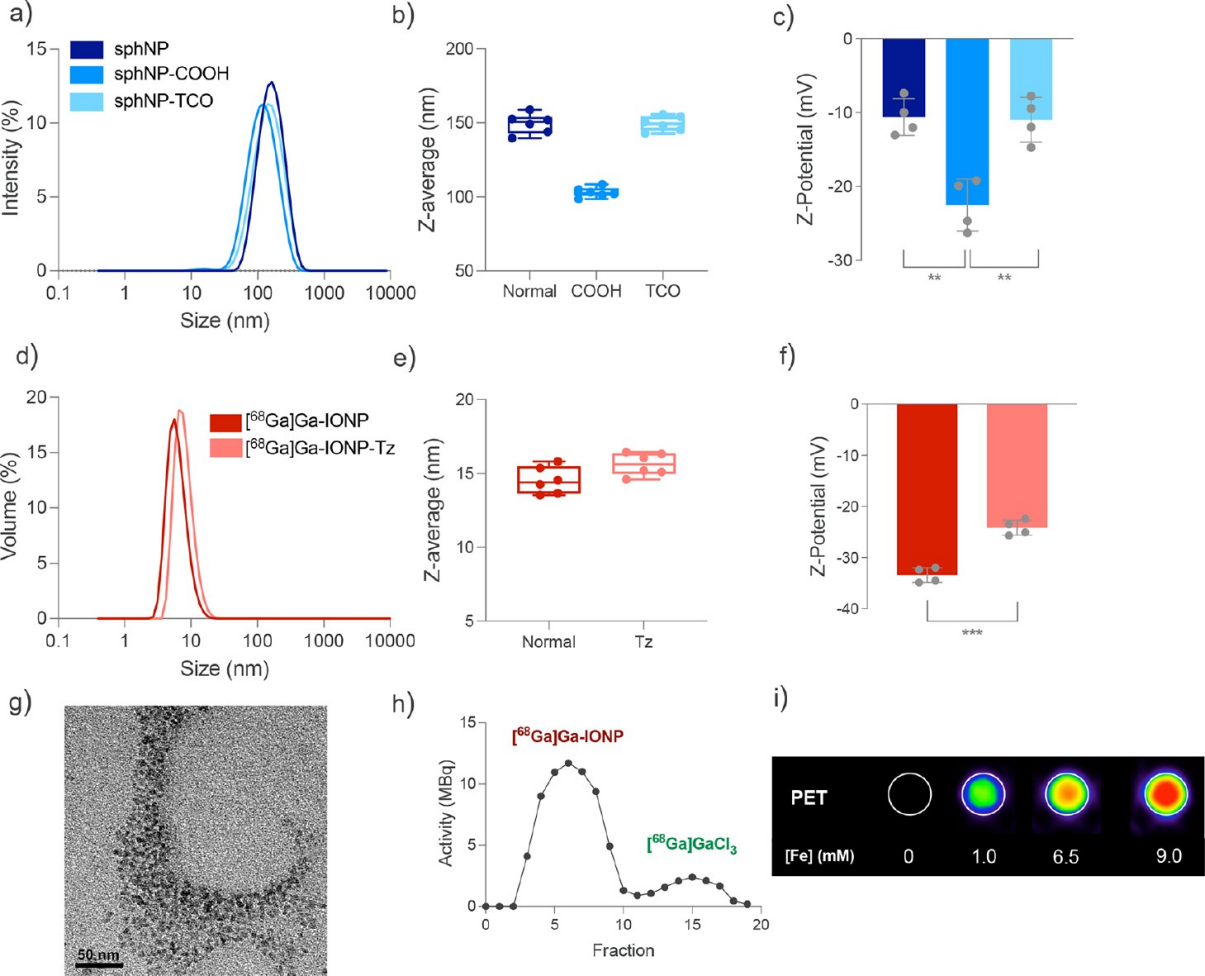
Physicochemical characterization of nanoparticles used for the pretargeting approach. (a) Hydrodynamic size distributions of sphNP, 18:1 Dodecanyl PE-containing sphNP (sphNP-COOH), and sphNP functionalized with TCO (sphNP-TCO) by DLS. (b) Hydrodynamic size values (z-average, mean ± SD) for six independent syntheses of sphNP, sphNP-COOH, and sphNP-TCO in H_2_O. (c) ζ-Potential values (mean ± SD) of four independent syntheses of sphNP, sphNP-COOH, and sphNP-TCO in H_2_O (statistical analysis by two-tailed *t* test; error bars indicate SD, *N* = 4; **p = 0.0015 (sphNP vs sphNP-COOH), **p = 0.0025 (sphNP-COOH vs sphNP-Tz)). (d) Hydrodynamic size distributions of [^68^Ga]­Ga-IONP and [^68^Ga]­Ga-IONP functionalized with Tz by DLS. (e) Hydrodynamic size values (z-average, mean ± SD) of six independent syntheses of [^68^Ga]­Ga-IONP and [^68^Ga]­Ga-IONP-Tz. (f) ζ-Potential values (mean ± SD) of four independent syntheses of [^68^Ga]­Ga-IONP and [^68^Ga]­Ga-IONP-Tz (statistical analysis by two-tailed *t* test; error bars indicate SD, *N* = 4; ***p = 0,0001). (g) Representative TEM image of [^68^Ga]­Ga-IONP. (h) Gel filtration radio-chromatogram of [^68^Ga]­Ga-IONP. (i) PET phantoms obtained at different iron and [^68^Ga]­Ga^3+^ concentrations of [^68^Ga]­Ga-IONP-Tz.

#### ^68^Ga-Doped Citrate-Coated IONP Functionalized with Tetrazine

[^68^Ga]­Ga-IONP functionalized with tetrazine were produced using a method previously described by our group.^12^ Briefly, extremely small iron oxide nanoparticles core-doped with ^68^Ga and stabilized using citrate were rapidly produced using a microwave-assisted synthesis method and purified by size-exclusion chromatography, taking only approximately 15 min to obtain a pure, ready-to-use sample. Subsequently, the tetrazine moiety was incorporated into the [^68^Ga]­Ga-IONP surface through amide formation between benzylamine tetrazine and the carboxylic acid groups of citrate, forming [^68^Ga]­Ga-IONP-Tz. The nanoparticles were analyzed to confirm that their properties matched those previously described.^15^ The results showed homogeneous size distributions with small hydrodynamic sizes of 14 ± 2 nm for [^68^Ga]­Ga-IONP (PDI = 0.23 ± 0.04) and 15 ± 1 nm for [^68^Ga]­Ga-IONP-Tz (PDI = 0.26 ± 0.03) (Figure 2d), matching previously obtained values. Furthermore, the method presented high reproducibility, showing minimal differences between the hydrodynamic sizes for the six independent syntheses (Figure 2e). The ζ-potential measurements were also similar to the published values, indicating the incorporation of tetrazine molecules by a shift in their surface charge from a mean value of −33 mV for [^68^Ga]­Ga-IONP to −24 mV for [^68^Ga]­Ga-IONP-Tz (*N* = 4) (Figure 2f). TEM analysis (*N* = 30 measured NP) determined a mean core size of 2.7 ± 0.3 nm for [^68^Ga]­Ga-IONP (Figure 2g), resembling prior studies. We calculated the radioactive elution profile of [^68^Ga]­Ga-IONP (values acquired from the size-exclusion chromatography step), which showed a large peak due to the ^68^Ga incorporated in the NP core and a small peak due to free [^68^Ga]­Ga^3+^ (Figure 2h). Considering that [^68^Ga]­Ga-IONP were designed for PET imaging, we tested their ability to provide PET signals using phantom images. The results indicated a concomitant rise in the PET signal with an increase in NP concentration (Figure 2i).

#### *In Vitro* Characterization of the Bioorthogonal Reaction

The incorporation of TCO and Tz molecules on the surface of the produced NP was suggested to be successful because of the shift in their surface charge, as measured by DLS. Nevertheless, to further demonstrate the presence of these moieties, we carried out fluorescence assays involving incubation of sphNP-TCO and IONP-Tz with fluorescently tagged complementary molecules Tz-Cy3 and TCO-Cy5, respectively. In addition, control solutions containing nonfunctionalized sphNP and IONP were incubated with Tz-Cy3 and TCO-Cy5, respectively. We observed that nonfunctionalized IONP after incubation and purification showed almost no fluorescence, but IONP-Tz showed a strong fluorescent signal indicating successful Tz ligation. Moreover, using a calibration curve, we calculated the amount of Tz molecules per mol of iron to be 10.1 ± 1.1 μmol Tz/mol Fe (*N* = 5). For sphNP, we were unable to obtain clear results because the dye adhered to both the control and TCO-functionalized SLNP, probably due to the nonspecific binding of sph molecules to the nanoparticle surface. Although the fluorescence was shown to behave differently between the two samples, we needed an additional method to demonstrate the presence of TCO molecules on the surface of the sphNP. Therefore, for this purpose, the bioorthogonal reaction was characterized using TEM.

To characterize the bioorthogonal reaction with TEM, sphNP-TCO was incubated with (a) IONP and (b) IONP-Tz for 2h at 37 °C. The resulting solutions were imaged using TEM (Figure 3), in which the large (approximately 100 nm) and low-contrast lipidic nanoparticles and very small (approximately 3 nm) and strong-contrast iron oxide nanotracers could easily be observed. The TEM analysis showed clear differences depending on the Tz-conjcugation of the IONP (Figure 3b). Nonfunctionalized IONP were randomly distributed around the lipidic nanoparticles and elsewhere and did not appear to be linked to the TCO-conjugated sphNP. In contrast IONP-Tz completely coated the surface of TCO-conjugated sphNP. These results confirmed the presence of TCO molecules on the surface of the sphNP and of Tz molecules on the surface of the IONP. Furthermore, this experiment confirmed the viability of bioorthogonal reactions *in vitro*.

**Figure 3:**
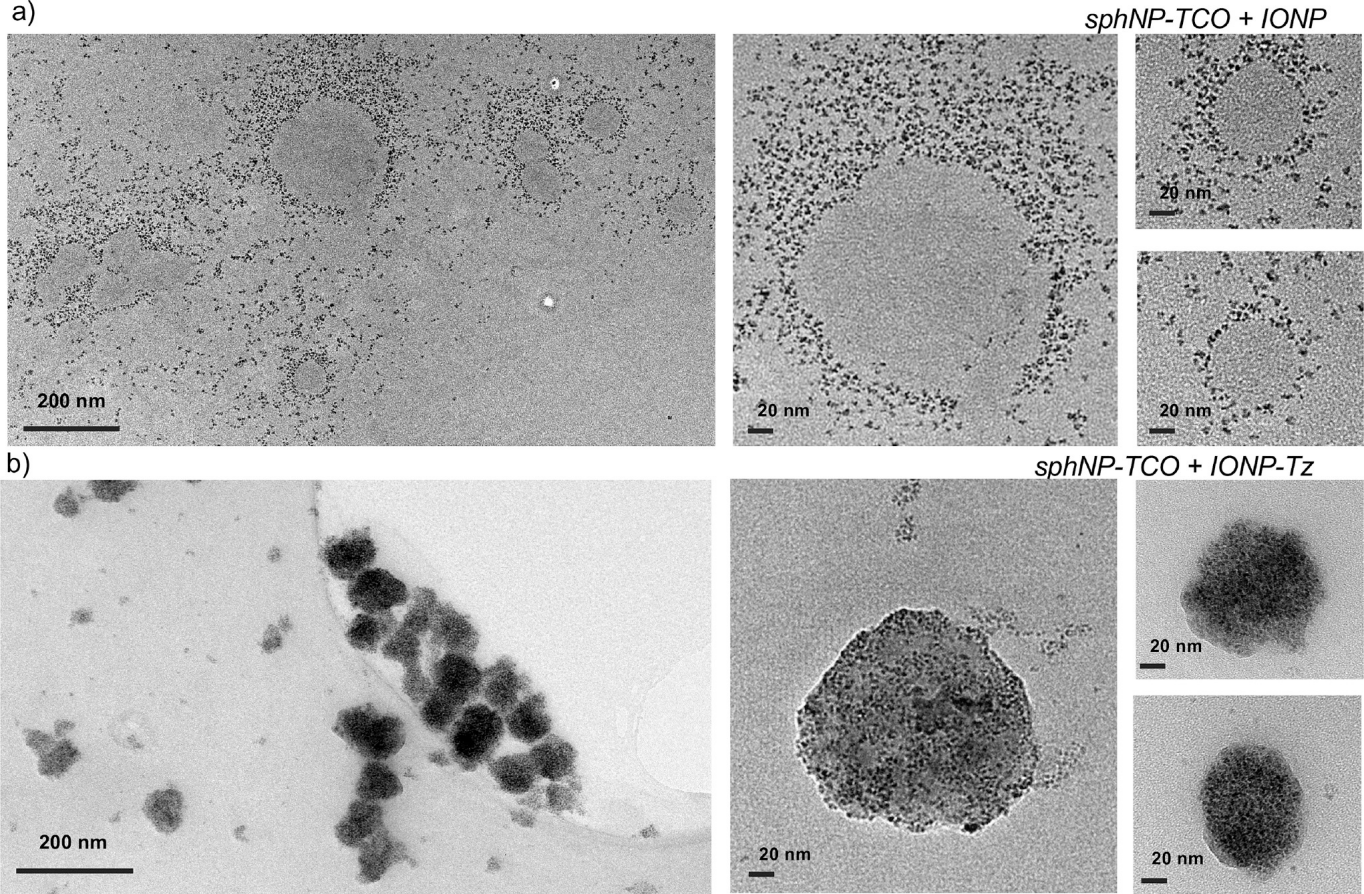
*In vitro* characterization of the bioorthogonal reaction using TEM. (a) TEM image of several TCO-conjugated sphNP surrounded by IONP (left); scale bar, 200 nm. Higher-magnification TEM images showing a single sphNP surrounded by but not binding to the nonfunctionalized IONP (small black particles) (right); scale bars, 20 nm. (b) TEM images of several sphNP-TCOs covered with IONP-TZs (left); scale bar, 200 nm. Higher magnification TEM images showing a single sphNP completely covered with IONP (small black particles) (right); scale bars, 20 nm.

#### *Ex Vivo* Analysis of the Pretargeting Method

After comprehensive characterization of the nanoparticles for the pretargeting approach and analysis of the bioorthogonal reaction performance *in vitro*, we continued with the *in vivo* experiments. Prior to performing the PET experiments, we carried out a “cold” (i.e., radiation-free) pilot experiment using fluorescence. The main goals of this experiment were to characterize the performance of the *in vivo* bioorthogonal reaction *ex vivo* and to evaluate whether the NP-to-NP approach could be successfully used for atherosclerosis detection. For this experiment, Ldlr^*–/–*^ mice (*N* = 30) were fed with HFD for 24 weeks and then divided into six groups intravenously injected with different combinations of NP. Mice from group 1 (*N* = 3) were injected with fluorescence-labeled sphNP-TCO that were allowed to circulate for 24 h prior to the end point. The goal of this group was to verify that the TCO molecules on the surface of SLNP did not hinder their ability to accumulate in atherosclerotic lesions. Mice from groups 2 (*N* = 6) and 3 (*N* = 6) were injected with sphNP-TCO, whereas mice from groups 4 (*N* = 6) and 5 (*N* = 6) were injected with sphNP. Alexa Fluor 647-labeled IONP-Tz was then injected intravenously into the mice from groups 2–5 to, either after 4 h (groups 2 and 4) or 24 h (groups 3 and 5), and allowed to circulate for 2 h prior to the end point. Finally, mice from group 6 (*N* = 3) were not injected, acting as controls. Groups 2 and 3 were designed to represent the full pretargeted approach with two different circulation times for the sphNP, thus allowing us to define the optimal time point for IONP injection. In addition, Groups 4 and 5 were designed as bioorthogonal reaction controls for Groups 2 and 3, respectively. At the end point, mice were euthanised by exsanguination and perfusion-fixed, and aortas and livers were harvested for analysis.

*Ex vivo* fluorescence of whole aortas (one aorta per group) was performed for proof of concept (Figure 4). The fluorescence level in all aortas was adjusted postacquisition to eliminate tissue autofluorescence using the noninjected control group (group 6) as a negative control. The aorta from mice injected only with the fluorescent sphNP-TCO showed a high-intensity signal, indicating that the ability of the sphNP to accumulate in atherosclerotic lesions was not impeded by the functionalization step. Furthermore, mice representing the full pretargeted approach with sphNP-TCO and A647-IONP-Tz (groups 2 and 3) showed fluorescent signals, but no signal was observed in the aortas from the control mice injected with sphNP and A647-IONP-Tz (groups 4 and 5). Moreover, when comparing aortas from groups 2 and 3, we observed that aortas from mice in which sphNP were allowed to circulate for 24 h showed higher intensity than aortas from mice in which sphNP were allowed to circulate only for 4 h. These results suggest the feasibility of the bioorthogonal reaction *in vivo*, while indicating bioorthogonal reaction specificity, since fluorescent IONP-Tz were shown to accumulate only in the aortas of mice injected with sphNP-TCO. Moreover, the results indicated that it would be more effective to allow the sphNP to circulate for 24 h rather than 4 h. Finally, the presence of a fluorescent signal in the aorta indicated that the pretargeting approach could be useful for atherosclerosis detection.

**Figure 4:**
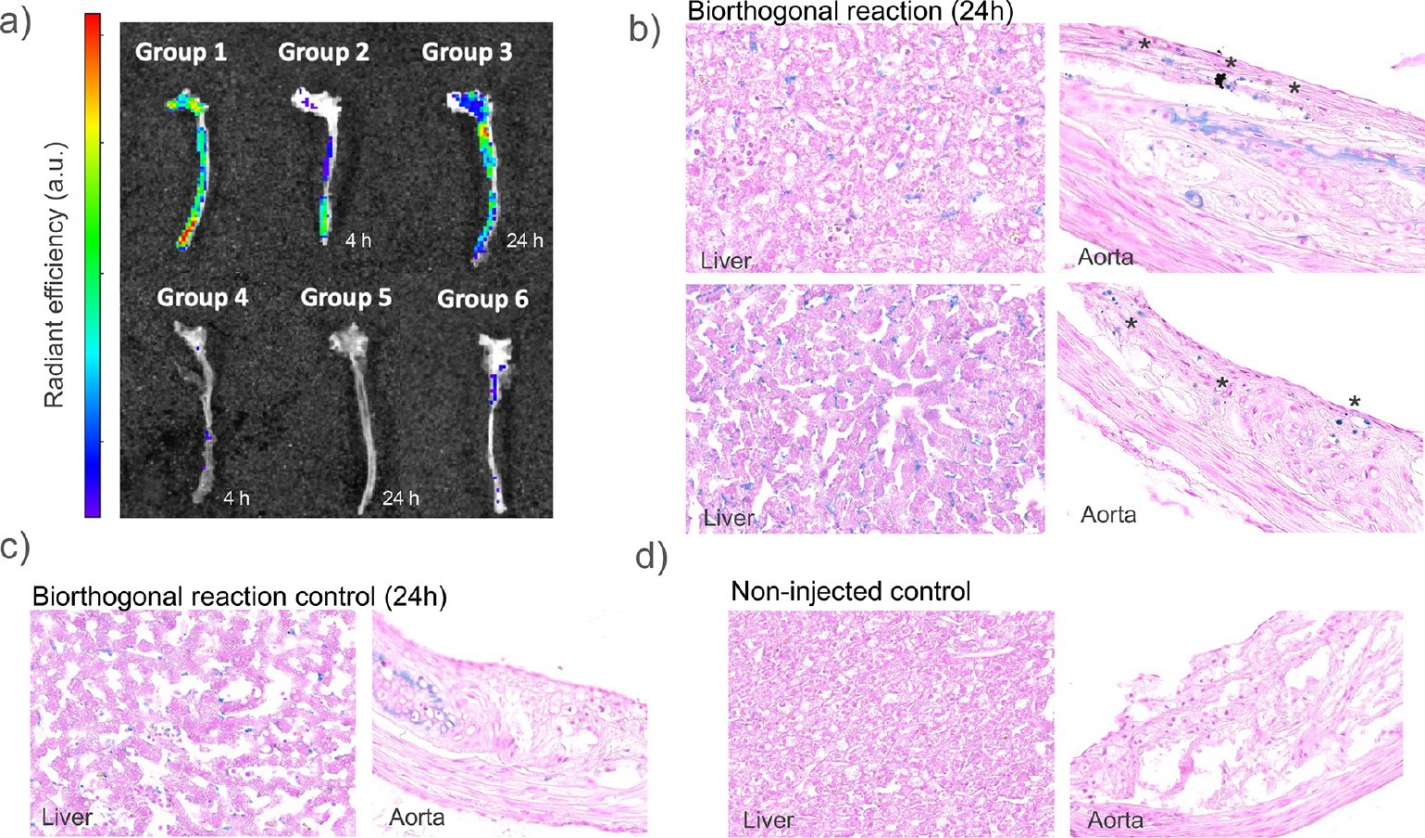
*Ex vivo* analysis of the pretargeting method by fluorescence imaging. The Ldlr^–/–^ mice (*N* = 30) were divided into six groups. Group 1 mice (*N* = 3) were injected with DiD-labeled sphNP-TCO. Groups 2 (*N* = 6) and 3 (*N* = 6) mice were injected with sphNP-TCO, which was allowed to accumulate for 4 and 24 h, respectively. Groups 4 (*N* = 6) and 5 (*N* = 6) mice were injected with sphNP, which were allowed to accumulate for 4 and 24 h, respectively. All mice from groups to 2–5 were then i.v. injected with A647-IONP-Tz. Mice in group 6 (*N* = 3) were not injected. (a) IVIS imaging of one aorta from each experimental group; levels were adjusted to show only signals exceeding tissue autofluorescence using the noninjected control group (group 6) as a negative control (one animal per group was randomly chosen for this analysis). (b–d) Representative Prussian blue-stained sections from the inner arch of the ascending aorta and liver of Ldlr^–/–^ mice. Sections of mice corresponding to (b) group 3: full pretargeted approach with IONP injection after 24h of sphNP circulation, (c) group 5: bioorthogonal reaction control with IONP injection after 24h of sphNP circulation, and (d) group 6: noninjected mice. The Prussian blue signal is shown in blue. Asterisks depict Prussian blue signals corresponding to IONP in the aorta.

For confirmation of the promising results from the fluorescence imaging experiments, the inner arch of the ascending aorta and the liver of the injected mice from groups 3, 5, and 6 were sectioned and stained with Prussian blue for iron visualization. Representative images of the histochemistry results are shown in Figure 4. The acquired images showed the presence of iron (blue spots) in the atherosclerotic plaques of the mice injected with the full pretargeted approach (group 3) (Figure 4b), whereas no punctate Prussian blue staining was observed in the plaques of the control mice groups (groups 5 and 6) (Figure 4c,d). Furthermore, iron stains were found in all the livers of mice injected with IONP, regardless of whether they belonged to the control or noncontrol groups (Figures 4b,c), while no staining was observed in the livers of the noninjected mice (Figure 4d). These results indicated that the mice were successfully injected with IONP, confirming that the lack of NP in the aortas of group 5 mice was not due to an injection failure, but rather due to the lack of the TCO moiety in the NP. Therefore, the histochemical results corroborated the observations from the fluorescence images. The combined findings indicated that the NP-to-NP pretargeting approach can deliver IONP into atherosclerosis lesions within 2 h thereby potentially enabling PET imaging with short half-life radioisotopes.

#### *In Vivo* PET Imaging

To test the applicability of the sphNP-IONP pretargeting approach in atherosclerosis PET imaging, we synthesized and functionalized radioactive [^68^Ga]­Ga-IONP or [^68^Ga]­Ga-IONP-Tz using a benchtop ^68^Ge/^68^Ga generator. Atherosclerosis was induced in Ldlr^*–/–*^ mice (*N* = 17) by 24 weeks of HFD feeding, which were subsequently divided into three groups. Group 1 (*n* = 7) received the full pretargeted approach, consisting of sphNP-TCO (i.v.) followed by [^68^Ga]­Ga-IONP-Tz (i.v.) after 24 h. Group 2 (*n* = 7) received sphNP-TCO (i.v.) followed by nonfunctionalized [^68^Ga]­Ga-IONP after 24 h to test the importance of the bio-orthogonal reaction. Group 3 (*n* = 3) received only [^68^Ga]­Ga-IONP-Tz, to test for nonspecific binding of IONP (Figure 5a).

**Figure 5:**
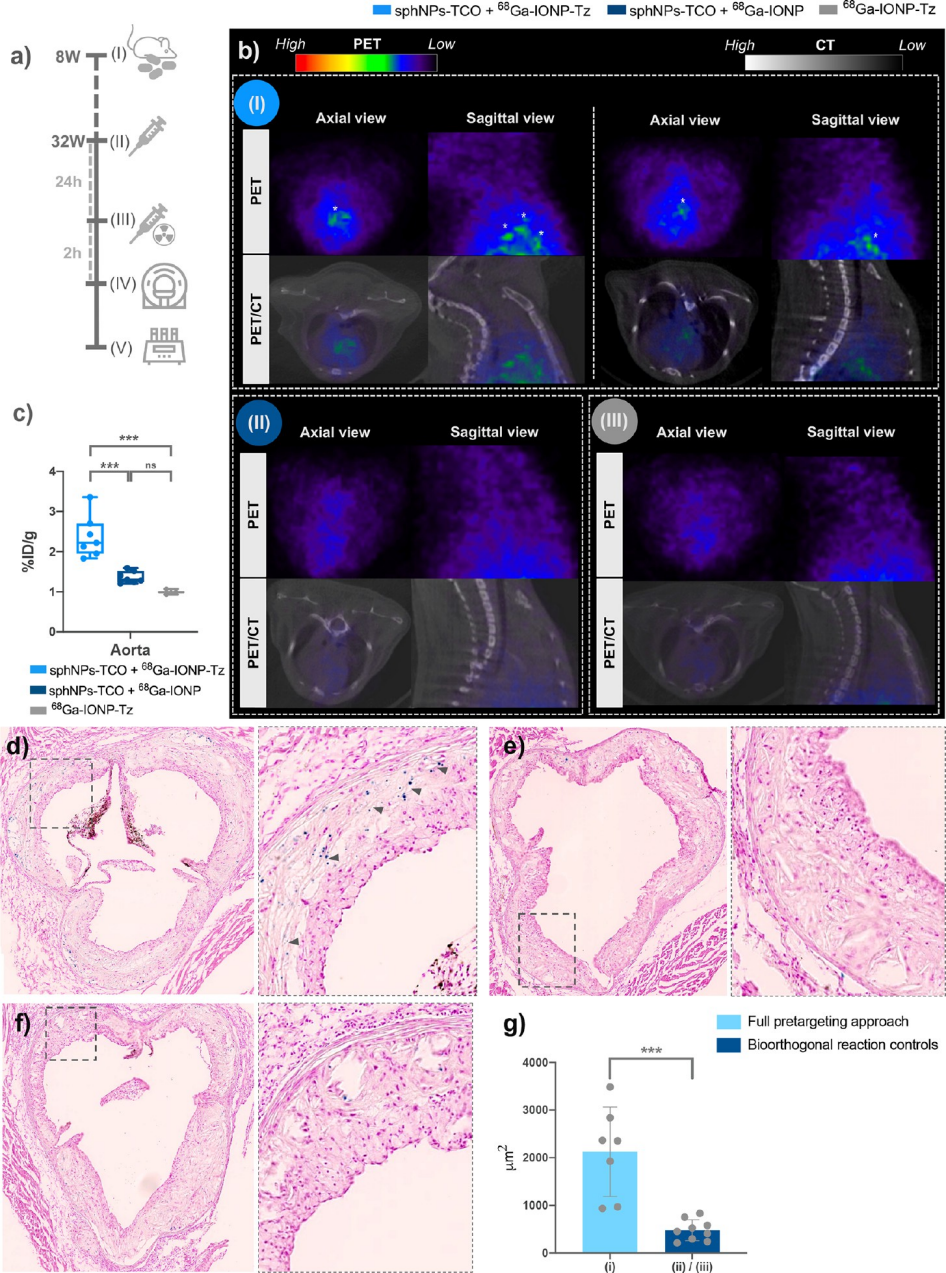
PET/CT images of the aortic arch after NP-to-NP pretargeting. (a) Experimental design; (b) representative aortic arch-focused PET/CT images of mice from (I) group 1 with the full pretargeted approach (sphNP-TCO + [^68^Ga]­Ga-IONP-Tz), (II) group 2 lacking Tz-functionalization of the [^68^Ga]­Ga-IONP to provide a bioorthogonal reaction control (sphNP-TCO + [^68^Ga]­Ga-IONP), and (III) group 3 receiving only [^68^Ga]­Ga-IONP-Tz. (*) indicates [^68^Ga]­Ga-IONP uptake. (c) Percentage of injected dose per gram of tissue (%ID/g) in the aorta per group. Each point represents a single mouse. The differences between the noncontrol group and the two control groups were significant (***p < 0.001). The differences between the control groups were not significant (ns, *p* = 0.323). (d–f) Representative Prussian blue-stained aortic root sections from Ldlr^–/–^ mice in (d) Group 1, (e) Group 2, and (f) and Group 3. The Prussian blue signal is shown in blue. Scale bars 400 μm. (g) Differences between the total Prussian blue area present in the atherosclerotic plaques of aortic root sections of mice from group 1 (full pretargeted approach) and bioorthogonal reaction control mice in groups 2 and 3. Each point represents the mean ± SD value of the total Prussian blue area measured in the plaque of the aortic root sections at different heights. Each point represents a single mouse. A two-tailed *t* test showed a statistically significant difference between the two groups (***p = 0.0004).

All mice were injected with ∼10 MBq of activity. After 2 h of circulation, *in vivo* whole-body PET scans (30 min) were acquired, followed by a CT scan (15 min) for anatomical reference. Animals were euthanised by CO_2_ exposure just after finishing the imaging studies, immediately exsanguinated, and slowly perfused through the left ventricle with 10 mL of 4% phosphate-buffered formaldehyde to avoid blood clotting. The organs (heart, aorta, liver, spleen, lungs, bladder, kidneys, bone, and muscle) were quickly extracted and rinsed in sterile 1× PBS to eliminate any blood contamination. Organ radioactivity was measured *ex vivo* using a γ counter. Finally, once the radioactivity from the tissues had decayed, the aortic roots were sectioned and stained with Prussian blue for analysis of the iron content in the plaques.

Whole-body PET/CT images of mice from the three different groups are shown in Figure S2.

No NP circulation was seen 2 h after nanoradiotracer injection. In addition, [^68^Ga]­Ga-IONP, both functionalized and nonfunctionalized, showed a similar biodistribution *in vivo*. As expected, all animals showed high NP accumulation in the liver, where they gathered before excretion. Furthermore, given the small size of IONP, some were eliminated by renal filtration; therefore, a small accumulation was observed in the bladder. Uptake in the aortic arch, where most atherosclerosis in mice develops, was evident only in the group with the full pretargeted approach, whereas no uptake was observed in the control mice (Figure S2).

Aortic arch-focused images from whole-body PET/CT scans, displaying a closer view of aortic arch uptake, are shown in Figure 5. Uptake spots depicting the shape of the aortic arch were clearly observed in this area in the images of mice from group 1 (Figure 5b-I). In contrast, images from groups 2 and 3did not show any noticeable signals in the aortic arch (Figure 5b-II,III). These results suggest that the pretargeted approach is capable of successfully detecting atherosclerotic lesions *in vivo*. Furthermore, they indicated that the accumulation of [^68^Ga]­Ga-IONP-Tz was due to the specificity of the bioorthogonal reaction with sphNP-TCO, since no uptake was observed in the two control conditions.

#### *Ex Vivo* Biodistribution Studies

Following the analysis of the PET scans, we evaluated the results obtained using a γ counter. The acquired data were decay-corrected and presented as the percentage of injected dose per gram of tissue (%ID/g). Plots represent the differences between groups 1, 2, and 3 in %ID/g for each extracted tissue (Figures 5c and S3).

The results showed large activity accumulations in the spleen and liver, typical of [^68^Ga]­Ga-IONP and NP size,^12^ whereas lower activity concentrations were observed in the rest of the organs. One-way ANOVAs were performed to analyze differences between mouse groups in the %ID/g of each organ. Significant differences were observed only in the aorta (Figure 5d). Furthermore, while the differences between the full pretargeted approach group and the two control groups were highly significant, the differences between the control groups were not significant. These results corroborated the clear uptake observed in the imaging experiments, while further indicating the specificity of the bioorthogonal reaction, showing only uptake in the aortas from mice injected with the TCO functionalized targeting NP (sphNP-TCO).

Finally, to complement the *in vivo* PET and *ex vivo* biodistribution studies, we analyzed iron content in atherosclerotic plaques using Prussian blue staining. Representative images of stained aortic root sections from mice in groups 1, 2, and 3 are shown in Figure 5d–f. IONP accumulation (blue spots) was clearly observed in the atherosclerotic plaques of sections from group 1 mice, whereas little accumulation was observed in the atherosclerotic plaques of sections of mice from groups 2 and 3. In addition, we quantified the total Prussian blue-stained area in the atherosclerotic plaques of the aortic root sections (mean ± SD, at three different heart levels) and found a statistically significant difference between group 1 and the control mice in groups 2 and 3 (***p = 0.0004, two-tailed *t* test, Figure 5g). These results confirmed our previous observations, indicating the specificity of the bioorthogonal reaction, given that only mice representing the full pretargeted approach in group 1 showed IONP accumulation in atherosclerotic lesions. These results further proved the uptake observed in the imaging experiments, confirming that the NP-to-NP pretargeted approach can successfully detect atherosclerosis *in vivo*.

## Conclusions

Here, we developed a novel concept for pretargeted imaging: nanoparticle-to-nanoparticle pretargeted imaging, in which no antibody is used and biological targeting is provided by a nanomaterial. We applied this concept for the *in vivo* characterization of atherosclerosis.

Solid lipid nanoparticles are among the most recently developed lipid nanoparticles and have emerged as promising candidates for theranostics. In our study, we hypothesized that sphNP would accumulate in the atherosclerosis region. The rationale for this assumption was based on (1) their expected long circulation times, which would facilitate their penetration into atherosclerotic plaques, (2) their composition, which would render them susceptible to sphingomyelinase degradation, and (3) our previous work on iron-oxide based sph nanomicelles. Regarding the use of sphNP as a targeting agent, the simplicity and reproducibility of the synthesis method, together with their demonstrated ability to accumulate in plaques, made them good candidates for pretargeting. Furthermore, it is a cost-effective method compared to antibody targeting, since the components of sphNP are cheap and can be more easily scaled for mass production. Overall, we demonstrated that the proposed pretargeting approach can be used to visualize atherosclerotic lesions in Ldlr–/– mice using PET. In addition, this method expands the use of bioorthogonal imaging using an NP-to-NP pretargeting approach rather than conventional antibody-based imaging. In addition to offering imaging possibilities, the characteristic properties of sphNP (such as biocompatibility, high stability, and lipid structure), together with their ability to accumulate in atherosclerotic lesions, render them suitable candidates for targeted drug delivery.

## Experimental Section

[^68^Ga]­GaCl_3_ (*t*_1/2_ = 68 min, β+ = 89%, and EC = 11%) was obtained from a ^68^Ge/^68^Ga generator system (ITG Isotope Technologies Garching GmbH, Germany) in which ^68^Ge (*t*_1/2_ = 270 days) was attached to a column based on an organic matrix generator. [^68^Ga]­GaCl_3_ was eluted with 4 mL of 0.05 M hydrochloric acid. Iron­(III) chloride, hydrazine monohydrate, *N*-(3-(dimethylamino)­propyl)-*N*′-ethylcarbodiimide hydrochloride, *N*-hydroxysulfosuccinimide sodium salt, trans-cyclooctene and tetrazine were purchased from Sigma-Aldrich. Citric acid trisodium salt dihydrate was purchased from Acros organics. Disposable PD-10 desalting salt columns were purchased from GE Healthcare Life.

### Sphingomyelin Solid Lipid Nanoparticles (sphNP)

#### Synthesis

For the synthesis of sphingomyelin solid lipid nanoparticles (sphNP), sph and cholesterol (Cho) were dissolved in EtOH at a concentration of 100 and 10 mg/mL, respectively. Subsequently, a mixture of 120 μL of sph solution and 100 μL of Cho solution was rapidly injected into 2 mL of Milli-Q H_2_O at 50 °C and under magnetic stirring. The solution was kept at those conditions for 3 min. The formed SLNPs were filtered by size exclusion chromatography, using PD-10 desalting columns to remove any lipid excess. The same protocol was followed for the synthesis of fluorescent sphNP except that 2.6 μL of DiIC18(5) fluorophore (25 mg/mL in EtOH) were added to the organic phase before injecting it into H_2_O. In addition, for the *in vivo* experiments, 55 mg of mannitol were added per ml of NPs to make them physiologically stable.

#### Functionalization with TCO

To produce trans-cyclooctene (TCO) functionalized sphNP a multiple step protocol was followed. First, sphNP that contained carboxyl groups (COOH) on their surface were prepared. For that purpose, a mixture of 120 μL of sph solution (100 mg/mL EtOH), 100 μL of Cho solution (10 mg/mL EtOH), and 20 μL of 1,2-dioleoyl-*sn*-glycero-3-phosphoethanolamine-*N*-(dodecanoyl) (18:1 Dodecanyl PE) solution (10 mg/mL EtOH) was rapidly injected into 2 mL of Milli-Q H_2_O at 50 °C and under magnetic stirring. The solution was kept at those conditions for 3 min. The formed SLNPs were filtered by size exclusion chromatography, using PD-10 desalting columns to remove any lipid excess. Upon completion of this step, the functionalization of their surface with TCO molecules was carried out. To do so, 0.05 mmol of *N*-(3-(dimethylamino)­propyl)-*N*′-ethylcarbodiimide hydrochloride (EDC) and 0.06 mmol of *N*-hydroxysulfosuccinimide sodium salt (Sulfo-NHS) were dissolved into 2.5 mL of sphNP-COOH to activate the surface carboxyl groups. The resulting solution was stirred for 30 min at r.t. followed by ultracentrifugation through 30 kDa Amicon filters to remove excess reagents (8000 rpm, 2 min, Hettich universal 320R centrifuge). Subsequently, SLNPs were resuspended in 1.5 mL of HEPES buffer (0.5 M, pH = 8) and 120 μL of TCO-amine hydrochloride salt (TCO-NH_2_) (1 mg/mL) were added to the solution. The mixture was stirred at r.t. for 60 min and then centrifuged again, under the same conditions, to remove the unreacted TCO. Finally, the obtained sphNP-TCO were resuspended in 2.5 mL of Milli-Q H_2_O. In addition, for the *in vivo* experiments, 55 mg of mannitol were added per ml of NPs to make them physiologically stable.

### Citrate-Coated Iron Oxide Nanoparticles (IONP)

#### Synthesis

For the synthesis of cit-IONPs, FeCl_3_·6H_2_O (75 mg) and citric acid trisodium salt (80 mg) were dissolved in Milli-Q H_2_O (9 mL). Subsequently, hydrazine monohydrate (1 mL) was added, and the mixture was rapidly introduced into the microwave (MW) (Anton Paar, GmbH73760, Ostfildern-Scharnhausen, Germany). Samples were ramped to 120 °C and held at this temperature for 10 min under vigorous stirring (240 W). Once this step was completed, the reaction mixture was cooled to 60 °C before it was purified through PD-10 desalting columns to eliminate unreacted species.

#### Radiolabeling Procedure

To produce radioactive [^68^Ga]­Ga^3+^ core-doped IONPs ([^68^Ga]­Ga-IONP), the same MW-driven synthesis used to produce IONPs was followed. However, in this protocol 2 mL of [^68^Ga]­Ga^3+^ chloride ([^68^Ga]­GaCl_3_) in HCL (0.05 M), were added to the aqueous mixture containing FeCl_3_·6H_2_O (75 mg) and citric acid trisodium salt (80 mg) dissolved in Milli-Q H_2_O (7 mL). Subsequently, hydrazine monohydrate (1 mL) was added, and the mixture was rapidly introduced into the microwave (MW) (Anton Paar, GmbH73760, Ostfildern-Scharnhausen, Germany). Samples were ramped to 120 °C and held at this temperature for 10 min under vigorous stirring (240 W). Once this step was completed, the reaction mixture was cooled to 60 °C before it was purified through PD-10 desalting columns to eliminate unreacted species, Radiolabeling yield was 83.9 ± 1.5.

##### Functionalization with Tetrazine

For the functionalization of the IONPs (2.5 mL) with tetrazine (Tz) first 0.07 mmol of EDC and 0.075 mmol of sulfo-NHS were dissolved into the NP solution, to activate the surface carboxyl groups provided by the citric acid. The mixture was stirred for 30 min at r.t. followed by ultracentrifugation through 30 kDa Amicon filters to remove any excess of reagents (8500 rpm, 3 min, Hettich universal 320R centrifuge). Subsequently, the NPs were resuspended in 1.5 mL of HEPES buffer (0.5 M, pH = 8) and 0.5 mg of benzylamine tetrazine hydrochloride, dissolved in 50 μL of dimethyl sulfoxide (DMSO), was added to the solution. The mixture was stirred for 60 min at r.t. and then centrifuged again, under the same conditions, to remove the unreacted Tz. The resulting NPs were resuspended in 2.5 mL of PBS.

### *In Vitro* Characterization of the Bioorthogonal Reaction

#### Fluorescence Assays

To characterize the amount of TCO and Tz present in the sphNP and the cit-IONPs respectively, two different fluorescence assays were carried out.

##### Quantification of TCO on the Surface of sphNP

A fluorescent probe Tz-Cy3 was used to calculate the amount of TCO in the surface of sphNP after conjugation. For this purpose, 1 mL of sphNP-TCO were mixed with 6 μL of Tz-Cy3 (1 mg/mL) and the resulting solution was incubated in a thermomixer at 37 °C for 120 min. Following this step, samples were filtered through PD-10 columns to remove the excess of fluorophore. Finally, the fluorescence signal of the filtered sample was measured (CLARIOstar plus, BMG Labtech, Germany) and compared with a previously constructed Tz-Cy3 calibration curve to obtain the concentration of TCO. Control solutions containing sphNP without TCO were also incubated with the fluorescent probe and analyzed in the same manner, to prove the reliability of this method.

##### Quantification of Tz on the Surface of IONP-Tz

A fluorescent probe, TCO-Cy5, was used to calculate the amount of Tz present on the surface of IONPs. For this purpose, 250 μL of IONP-Tz were mixed with 4 μL of TCO-Cy5 (1 mg/mL), and the resulting solution was incubated in a thermomixer at 37 °C for 120 min. Upon completion of this step, the sample was ultracentrifuged through 30 kDa Amicon filters to remove the excess of TCO-Cy5 (10,000 rpm, 3 min, Hettich universal 320R centrifuge). This step was repeated until the remainder of the filtration was clear. Finally, the fluorescence signal of the final sample was measured (CLARIOstar plus, BMG Labtech, Ortenberg, Germany), and compared with a previously constructed TCO-Cy5 calibration curve to obtain the concentration of Tz. Control solutions containing IONPs without Tz were also incubated with the fluorescent probe and analyzed in the same manner, to prove the reliability of this method.

### Animal Models

Animal experiments were approved by the ethical review boards at CNIC and Universidad Autónoma and permitted by the Comunidad de Madrid (PROEX020.8/21). Furthermore, all experiments followed the 3R principles to include the minimum number of animals required for sufficient statistical power. Transgenic mice (models described below) were used. All compared mice were littermates, housed together, and subjected to the same procedures.

### Low Density Lipoprotein Receptor Knockout (Ldlr^*–/–*^) Mice

Ldlr^–/–^ mice (B6.129S7-Ldlr^tm^1Her/J, the Jackson Laboratory) were used to study the accumulation of sphNP in plaques by confocal microscopy and noninvasive imaging. Ldlr^–/–^ mice have elevated plasma cholesterol level and develop atherosclerosis slowly on standard laboratory diet.^23^ On high-fat diets, they have very high plasma cholesterol levels and fast development of atherosclerotic plaques.^23^ Early lesions and advanced plaques were induced by feeding the mice for 24 weeks with standard laboratory diet (Rod18-A, SODISPAN) and high-fat diet (S9167-E011, Sniff), respectively. Only female mice were used.

### Positron Emission Tomography

For PET/CT acquisition, mice were intravenously injected with sphNP-TCO (150 μL). Following this, 24 h post sphNP injection, mice were intravenously injected with 8–10 MBq of [^68^Ga]­Ga-IONP-Tz (150 μL). After NP injection mice were anesthetised with 2% isoflurane and 1.8 L/min oxygen flow and positioned on a thermoregulated (37 °C) mouse bed with continuous monitoring of the respiratory cycle. Ophthalmic gel was placed in the eyes to prevent retinal drying.

*In vivo* PET/CT imaging in mice was performed with a nanoPET/CT small-animal imaging system (Mediso Medical Imaging Systems, Budapest, Hungary). List-mode PET data acquisition commenced 120 min after [^68^Ga]­Ga-IONP-Tz injection and continued for 30 min. At the end of PET, a micro-CT was performed, for attenuation correction and anatomic reference. The CT was acquired using an X-ray beam current of 178 μA and a tube voltage of 45 kVp and reconstructed using a RamLak algorithm. The PET images were reconstructed using Teratomo 3D algorithm with 6 subsets and 4 iterations, in an 80 × 80 × 170 matrix (voxel dimensions of 0.4 mm). Images were obtained and reconstructed with proprietary Nucline software (Mediso, Budapest, Hungary).

### Fluorescence Imaging

*Ex vivo* IVIS imaging (XENOGEN IVIS 200, PerkinElmer, Massachusetts) was performed to the extracted organs of Ldlr^*–/–*^ mice injected with fluorescent NPs. This technique was mainly used to image the livers to confirm that the NP injection was made correctly, and image the aortas to assess if there was any NP accumulation.

### Organs Biodistribution Analysis

The radioactivity of the blood and several tissues (muscle, bladder, kidney, liver, spleen, heart, lung, bone, and aorta) extracted from Ldlr^*–/–*^ mice, after animal perfusion, were measured using a γ-counter (Wizard 1470 PerkinElmer, Massachusetts), to study the accumulation of the [^68^Ga]­Ga-IONP in the different organs. The data acquired from this procedure was decay-corrected and presented as percentage of injected dose per gram of tissue (% ID/g).

### Tissue Processing and Staining

Tissues, including the heart, aorta, liver, spleen, lung, and kidney, were cryoprotected in sucrose solution (24 h in 25% sucrose followed by 24 h in 50% sucrose) followed by embedding in Tissue-Tek O.C.T. Compound (Sakura, Flemingweg, Netherlands) and snap freezing in liquid nitrogen. For microscopic analysis of atherosclerosis, ten cross sections (5 μm) of the ascending part of the extracted aorta were collected every 100 μm until reaching the brachiocephalic artery. Furthermore, the extracted heart was processed to obtain cross sections of the aortic root (5 μm) from the commissures of the aortic cusps upward. Finally, O.C.T. blocks containing multiple organs (liver, spleen, lung, and kidney) were prepared and cross sections (5 μm) that showed every organ in a single cut were obtained.

#### Histochemical Stains

Cross sections of the aortic root and the multiorgan blocks were stained with Prussian blue Prussian Blue to detect the presence of iron. Staining processes were carried out by the Histopathology Unit at CNIC.

### Confocal Microscopy

Confocal microscopy was used to analyze immunofluorescence-stained sections and to study the accumulation of fluorescent NPs. All fluorescent cross sections were analyzed with a Leica TCS SP5 microscope (Leica, Wetzlar, Germany) using either a HCX PL APO lambda blue 20*x*/0.7 multi-immersion objective or a HCX PL APO CS 40*x*/1.25 oil objective. Leica LAS X software was used for image acquisition. Furthermore, an acquisition method was designed for every experiment adjusting the laser intensities and the detection bandwidth to the required combinations. Moreover, the acquisition method was kept constant for every cross-section belonging to the same experiment so that comparisons between images could be performed.

### PET Scans Analysis

PET scan images were analyzed qualitatively using Horos Project software (New York) by looking for spots with high intensity signal in the aorta. All experiments included control groups used to compare images and demonstrate the reliability of the method.

#### Prussian Blue Prussian Blue Quantification

Prussian blue staining in the aortic roots of Ldlr^*–/–*^ mice injected with cit-IONPs were analyzed quantitatively using Fiji (Fiji is just ImageJ (Maryland)). To do so, images were split in different color channels, using the Color Deconvolution Plugin with the provided “Alcian blue & H” vector. Following this, a region of interest (ROI) corresponding to total plaque area was defined manually. Finally, total Prussian blue area was measured within the previously defined ROI by pixel thresholding segmentation, using an automatic threshold (MaxEntropy).

### Statistical Analysis

Statistical tests were performed in Prism 8 (GraphPad Software). Two-sample comparisons were analyzed by the unpaired Student’s *t* test for normally distributed data and by Mann–Whitney test for non-normally distributed data. Three or more samples’ comparisons were performed with the ordinary one-way ANOVA test for normally distributed data and by the Kruskal–Wallis test for non-normally distributed data. Moreover, normally distributed data with significantly different SD were analyzed using the Brown-Forsythe ANOVA test. For data that involved the effect of two independent variables, e.g., treatment and time, a mixed effects model was used. All tests were 2-tailed, and differences were considered statistically significant at *p* < 0.05. Bars in scatter dot plots represent mean ± SD.

## Supplementary Material



## Notes

Additional PET/CT images and biodistribution analysis, experimental details (PDF)

## Notes

In memory of Dr. Irene Fernández-Barahona.

## Abbreviations

PET: positron emission tomography
CT: computed tomography
IONP: iron oxide nanoparticles
EDC: *N*-(3-(dimethylamino)­propyl)-*N*′-ethylcarbodiimide hydrochloride
NHS: *N*-hydroxysuccinimide
DLS: dynamic light scattering
TEM: transmission electron microscopy
sph: sphingomyelin
sphNP: sphingomyelin solid lipid nanoparticles

## References

[1] Sletten E. M., Bertozzi C. R. (2009). Bioorthogonal Chemistry: Fishing for Selectivity in
a Sea of Functionality. Angew. Chem., - Int.
Ed..

[2] Carroll L., Evans H. L., Aboagye E. O., Spivey A. C. (2013). Bioorthogonal Chemistry
for Pre-Targeted Molecular Imaging-Progress and Prospects. Org. Biomol. Chem..

[3] Zeglis B. M., Sevak K. K., Reiner T., Mohindra P., Carlin S. D., Zanzonico P., Weissleder R., Lewis J. S. (2013). A Pretargeted PET
Imaging Strategy Based on Bioorthogonal Diels-Alder Click Chemistry. J. Nucl. Med..

[4] Yan C., Wu Y., Feng J., Chen W., Liu X., Hao P., Yang R., Zhang J., Lin B., Xu Y., Liu R. (2013). Anti-Avβ3
Antibody Guided Three-Step Pretargeting Approach
Using Magnetoliposomes for Molecular Magnetic Resonance Imaging of
Breast Cancer Angiogenesis. Int. J. Nanomed..

[5] Borén J., Chapman M. J., Krauss R. M., Packard C. J., Bentzon J. F., Binder C. J., Daemen M. J., Demer L. L., Hegele R. A., Nicholls S. J., Nordestgaard B. G., Watts G. F., Bruckert E., Fazio S., Ference B. A., Graham I., Horton J. D., Landmesser U., Laufs U., Masana L., Pasterkamp G., Raal F. J., Ray K. K., Schunkert H., Taskinen M.-R., van de Sluis B., Wiklund O., Tokgozoglu L., Catapano A. L., Ginsberg H. N. (2020). Low-Density Lipoproteins Cause Atherosclerotic
Cardiovascular Disease: Pathophysiological, Genetic, and Therapeutic
Insights: A Consensus Statement from the European Atherosclerosis
Society Consensus Panel. Eur. Heart J..

[6] Borén J., Williams K. J. (2016). The Central Role of Arterial Retention of Cholesterol-Rich
Apolipoprotein-B-Containing Lipoproteins in the Pathogenesis of Atherosclerosis:
A Triumph of Simplicity. Curr. Opin. Lipidol..

[7] Tabas I., Williams K. J., Borén J. (2007). Subendothelial Lipoprotein Retention
as the Initiating Process in Atherosclerosis. Circulation.

[8] Devlin C. M., Leventhal A. R., Kuriakose G., Schuchman E. H., Williams K. J., Tabas I. (2008). Acid Sphingomyelinase
Promotes Lipoprotein
Retention Within Early Atheromata and Accelerates Lesion Progression. Arterioscler., Thromb., Vasc. Biol..

[9] Zalewski A., Macphee C. (2005). Role of Lipoprotein-Associated
Phospholipase A 2 in
Atherosclerosis. Arterioscler., Thromb., Vasc.
Biol..

[10] Muñoz-Hernando M., Nogales P., Fernández-Barahona I., Ruiz-Cabello J., Bentzon J. F., Herranz F. (2024). Sphingomyelinase-Responsive Nanomicelles
for Targeting Atherosclerosis. Nanoscale.

[11] Lechuga-Vieco A. V., Groult H., Pellico J., Mateo J., Enríquez J. A., Ruiz-Cabello J., Herranz F. (2018). Protein Corona and Phospholipase
Activity Drive Selective Accumulation of Nanomicelles in Atherosclerotic
Plaques. Nanomed.: Nanotechnol. Biol. Med..

[12] Pellico J., Ruiz-Cabello J., Saiz-Alía M., del Rosario G., Caja S., Montoya M., de Manuel L. F., Morales M. P., Gutiérrez L., Galiana B., Enríquez J. A., Herranz F. (2016). Fast Synthesis and Bioconjugation of 68 Ga Core-Doped
Extremely Small Iron Oxide Nanoparticles for PET/MR Imaging. Contrast Media Mol. Imaging.

[13] Scinto S. L., Bilodeau D. A., Hincapie R., Lee W., Nguyen S. S., Xu M., Ende C. W. A., Finn M. G., Lang K., Lin Q., Pezacki J. P., Prescher J. A., Robillard M. S., Fox J. M. (2021). Bioorthogonal Chemistry. Nat.
Rev. Methods Primer.

[14] Choi J. Y., Lee B. C. (2015). Click Reaction: An Applicable Radiolabeling
Method
for Molecular Imaging. Nucl. Med. Mol. Imaging.

[15] Pellico J., Fernández-Barahona I., Benito M., Gaitán-Simón Á., Gutiérrez L., Ruiz-Cabello J., Herranz F. (2019). Unambiguous Detection of Atherosclerosis using Bioorthogonal
Nanomaterials. Nanomed.: Nanotechnol. Biol.
Med..

[16] Adrover J. M., Pellico J., Fernández-Barahona I., Martín-Salamanca S., Ruiz-Cabello J., Hidalgo A., Herranz F. (2020). Thrombo-Tag, an In
Vivo Formed Nanotracer for the Detection of Thrombi in Mice by Fast
Pre-Targeted Molecular Imaging. Nanoscale.

[17] Meyer J.-P., Houghton J. L., Kozlowski P., Abdel-Atti D., Reiner T., Pillarsetty N. V. K., Scholz W. W., Zeglis B. M., Lewis J. S. (2016). 18 F-Based Pretargeted
PET Imaging Based on Bioorthogonal
Diels–Alder Click Chemistry. Bioconjugate
Chem..

[18] Pellico J., Fernández-Barahona I., Ruiz-Cabello J., Gutiérrez L., Muñoz-Hernando M., Sánchez-Guisado M. J., Aiestaran-Zelaia I., Martínez-Parra L., Rodríguez I., Bentzon J., Herranz F. (2021). HAP-Multitag, a PET and Positive
MRI Contrast Nanotracer for the Longitudinal Characterization of Vascular
Calcifications in Atherosclerosis. ACS Appl.
Mater. Interfaces.

[19] Adrover J. M., Pellico J., Fernández-Barahona I., Martín-Salamanca S., Ruiz-Cabello J., Hidalgo A., Herranz F. (2020). Thrombo-Tag, an *in Vivo* Formed Nanotracer for the Detection of Thrombi in
Mice by Fast Pre-Targeted Molecular Imaging. Nanoscale.

[20] Pellico J., Fernández-Barahona I., Benito M., Gaitán-Simón Á., Gutiérrez L., Ruiz-Cabello J., Herranz F. (2019). Unambiguous Detection of Atherosclerosis Using Bioorthogonal
Nanomaterials. Nanomed.: Nanotechnol. Biol.
Med..

[21] Pellico J., Lechuga-Vieco A. V., Almarza E., Hidalgo A., Mesa-Nuñez C., Fernández-Barahona I., Quintana J. A., Bueren J., Enríquez J. A., Ruiz-Cabello J., Herranz F. (2017). In Vivo Imaging of
Lung Inflammation with Neutrophil-Specific 68Ga Nano-Radiotracer. Sci. Rep..

[22] Sloat B. R., Sandoval M. A., Li D., Chung W. G., Lansakara-P D. S. P., Proteau P. J., Kiguchi K., Digiovanni J., Cui Z. (2011). In Vitro and in Vivo Anti-Tumor Activities of a Gemcitabine Derivative
Carried by Nanoparticles. Int. J. Pharm..

[23] James M. L., Gambhir S. S. (2012). A Molecular Imaging Primer: Modalities,
Imaging Agents,
and Applications. Physiol. Rev..

[24] Mulder W. J. M., Jaffer F. A., Fayad Z. A., Nahrendorf M. (2014). Imaging and
Nanomedicine in Inflammatory Atherosclerosis. Sci. Transl. Med..

