# Supplementary material for: Nanoparticle to Nanoparticle Bioorthogonal Detection of Atherosclerosis
Source: ACS Appl Mater Interfaces. 2025 Oct 6;17(41):56675–86. doi: 10.1021/acsami.5c08945 (PMC12532092; doi:10.1021/acsami.5c08945)
Supplement: Supplementary file 1 [file am5c08945_si_001.pdf]

SUPPORTING INFORMATION

Nanoparticle to nanoparticle bioorthogonal detection of atherosclerosis

María Muñoz-Hernando[a,b], Paula Nogales[b], Marta Ibañez,[d] Miguel Ángel Morcillo,[d] Leticia González,[b] Jacob F. Bentzon[b,c], Fernando Herranz*[a,e]*

[a]Grupo de Nanomedicina e Imagen Molecular, Instituto de Química Médica (IQM/CSIC). Juan de la Cierva 3, 28006 Madrid, Spain. fherranz@iqm.csic.es

[b]Centro Nacional de Investigaciones Cardiovasculares, CNIC, Melchor Fernández-Almagro 3, 28029 Madrid, Spain.

[c]Department of Clinical Medicine, Aarhus University, Denmark.

[d]Unidad de Aplicaciones Médicas de las Radiaciones Ionizantes, Centro de Investigaciones Energéticas, Medioambientales y Técnicas (CIEMAT). Madrid. Spain.

[e]CIBER Enfermedades Respiratorias (CIBERES). Melchor Fernández-Almagro 3, 28029 Madrid, Spain.

In memory of Dr. Irene Fernández-Barahona

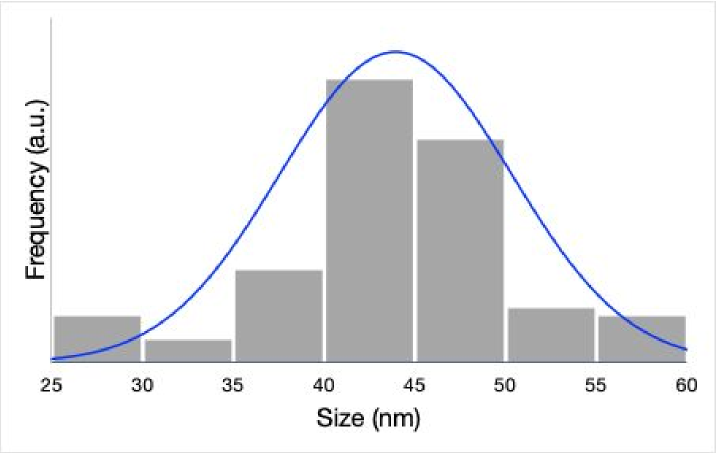

Figure S1. Particle size distribution for sphNP according to SEM images (N = 100).

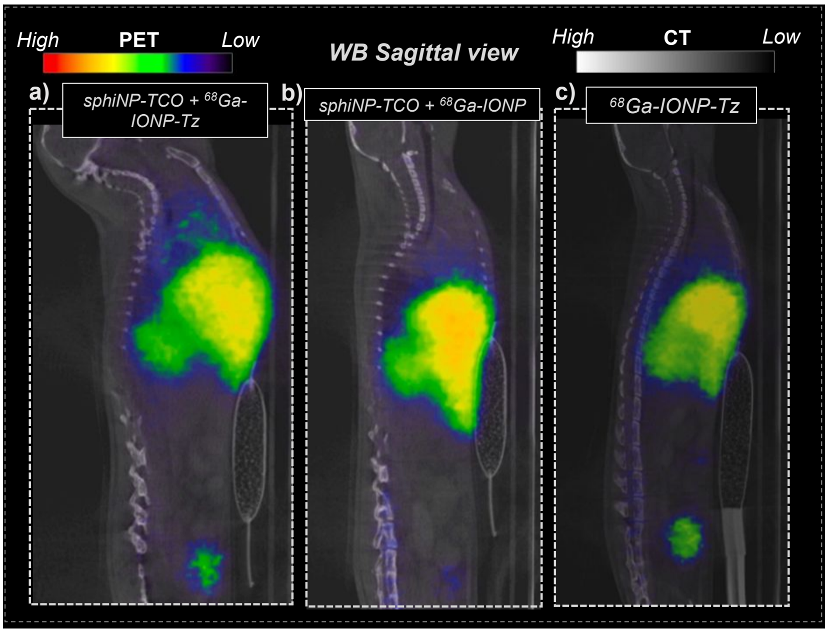

Figure S2. Whole body PET/CT scan images of the NP-to-NP pretargeting approach. Representative whole-body PET/CT scan sagittal images of mice from groups (a) (i) full pretargeted approach (sphiNP-TCO + ^{68}Ga-IONP-Tz), (b) (ii) bioorthogonal reaction control I (sphiNP-TCO + ^{68}Ga-IONP) and (c) (iii) bioorthogonal reaction control II (^{68}Ga-IONP-Tz). SUPP INFO

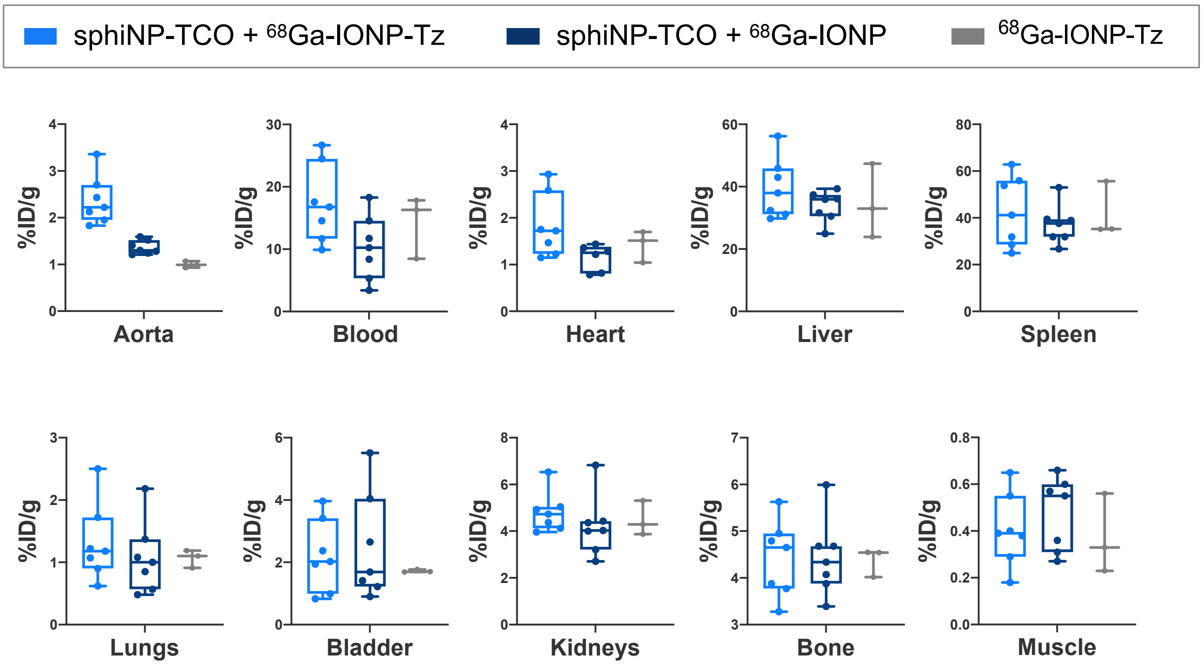

Figure S3. *Ex vivo* analysis of the pretargeting method by radioactivity biodistribution. Plots represent the differences on the percentage of injected dose per gram of tissue (%ID/g) for all the extracted organs from the mice of the experiment described in Figure 4.17. Each point represents one mouse. (Right) Results from one-way ANOVA of the aorta data. Differences between the non-control group and the two control groups were significant (***$p < 0.001$)

Physicochemical characterisation of nanoparticles

Dynamic light scattering (DLS)

The hydrodynamic size, polydispersity index (PDI), and zeta potential of all the synthesised NPs was measured by dynamic light scattering (DLS) (Zetasizer Nano ZS90, Malvern Instruments, Malvern, UK). All measurements were performed at r.t. and in triplicates. In addition, DLS was used to assess the colloidal stability of the NPs, by monitoring the changes in their hydrodynamic size and PDI with time, and after their incubation with different buffers (PBS, HEPES, Mannitol, and mouse serum).

Transmission electron microscopy (TEM)

The morphology and the core size of the NPs were determined by transmission electron microscopy (TEM). TEM analyses were carried out at the National Center of Electron Microscopy of the Universidad Complutense de Madrid (Madrid, Spain). For the magnetic NPs (SPHIONMs and cit-IONPs), a drop of diluted NP sample was placed on top of a carbon-coated copper grid and observed under TEM using a 200 keV JEOL- 2000 FXII instrument (Jeol Ltd., Tokyo, Japan). For the lipidic NPs (sphiNP) a drop of diluted sample was placed on the surface of a carbon-coated copper grid, negatively stained with 2% uranyl acetate, and observed under TEM using a 100 keV JEOL JEM 1400 instrument (Jeol Ltd., Tokyo, Japan).

Fourier transform infrared spectroscopy (FT-IR)

Fourier transform infrared spectroscopy (FT-IR) spectra were obtained for the SPHIONMs on a Perkin Elmer Spectrum 400 Series spectrometer (Perkin Elmer, USA); each spectrum was obtained by averaging 32 interferograms with a resolution of 1 cm^{-1}.

Thermogravimetric Analysis (TGA)

Thermogravimetric analysis (TGA) spectra of the SPHIONMs were obtained with a Seiko TG/ATD 320 U, SSC 5200 spectrometer (Seiko Instruments, Chiba, Japan) at the Universidad Autónoma de Madrid (Institute of Materials Science, Madrid, Spain). For this purpose, SPHIONMs were lyophilised, and the dried sample was heated from 20 ºC to 1000 ºC at 10 ºC/min under a 100 mL/min air flow.

Relaxometry

The longitudinal (T_1) and transverse (T_2) relaxation times of SPHIONMs at different concentrations were measured using a relaxometer at 1.5 T and 37 ºC (Bruker MQ60, Bruker Biospin, Germany). The obtained relaxation rates (R_1=1/T_1, R_2=1/T_2) were then plotted against their corresponding iron concentration values, to obtain the relaxivity values of the SPHIONMs.

Transmission electron microscopy

TEM images of samples containing the bioorthogonal reaction components, were acquired to demonstrate the specificity of the reaction *in vitro*. For this purpose, two different solutions were prepared. The first solution was composed of sphiNP-TCO (200 µL), IONPs-Tz (200 µL) and Milli-Q H_2O (200 µL), and the second one of sphiNP-TCO (200 µL), IONPs (200 µL), and Milli-Q H_2O (200 µL). Both solutions were incubated for 120 min at 37 ºC in a thermomixer to allow the bioorthogonal reaction to take place. Following this, samples were centrifuged through 100 kDa Amicon filters to remove unreacted NPs. The resulting solutions were imaged using a 200 keV JEOL- 2000 FXII transmission electron microscope (Jeol Ltd. Japan).
